# Embodied Intelligence in Soft Robotics Through Hardware Multifunctionality

**DOI:** 10.3389/frobt.2021.724056

**Published:** 2021-11-17

**Authors:** Matteo Cianchetti

**Affiliations:** ^1^ The BioRobotics Institute, Scuola Superiore Sant’Anna, Pontedera, Italy; ^2^ Department of Excellence in Robotics and AI, Scuola Superiore Sant’Anna, Pisa, Italy

**Keywords:** soft robotics, embodied intelligence, morphological computation, soft mechatronics, soft actuators

## Abstract

The soft robotics community is currently wondering what the future of soft robotics is. Therefore, it is very important to identify the directions in which the community should focus its efforts to consolidate its impact. The identification of convincing applications is a priority, especially to demonstrate that some achievements already represent an attractive alternative to current technological approaches in specific scenarios. However, most of the added value of soft robotics has been only theoretically grasped. Embodied Intelligence, being of these theoretical principles, represents an interesting approach to fully exploit soft robotic’s potential, but a pragmatic application of this theory still remains difficult and very limited. A different design approach could be beneficial, i.e., the integration of a certain degree of continuous adaptability in the hardware functionalities of the robot, namely, a “flexible” design enabled by hardware components able to fulfill multiple functionalities. In this paper this concept of flexible design is introduced along with its main technological and theoretical basic elements. The potential of the approach is demonstrated through a biological comparison and the feasibility is supported by practical examples with state-of-the-art technologies.

## Introduction

A widespread use of soft robotics technologies is highlighting the necessity of a different approach on the design of systems and sub-systems that can benefit from the properties of soft bodied parts to accomplish some specific. A peculiarity that plays a key role in the use of soft or compliant materials is their highly nonlinear mechanical behaviour. This is introducing complexity in modelling and usability, but it also represents a remarkable source of behavioural richness. The importance of body characteristics is also central in the new interpretation of Artificial Intelligence called Embodied Intelligence (EI). According to this theory, the body of an agent plays a fundamental role in simplifying tasks and in making adaptive behaviours emerge through the interaction with the environment (Pfeifer and Bongard, 2007). The body can serve as a computational means (Morphological Computation) and the way it affects the environmental interactions is determined through three fundamental aspects: material properties, shape and arrangement of its components. However, how to pragmatically implement EI principles remains elusive. Thus, on one side, technological development of soft mechatronics components is progressing, increasing the exploitation of all the advantages rising from intrinsic softness and compliance (as shown in the brief state of the art reported in *The Long Way Ahead for Soft Mechatronics Development* that also set the technological groundwork of the paper). On the other side, these technologies are necessary but may be not sufficient to create a transformational impact. EI represents an attractive design paradigm for soft robotic systems, but it is still difficult to apply. A step forward may be represented by a different design approach: not to formally describe and follow design rules to implement EI principles a priori, but rather to embed basic elements and tools with a “flexible” design approach (introduced in *Embodied Intelligence by Flexible Design*), and let the system evolve and adapt its body functionalities to exploit EI principles through the direct interaction with the environment. This evolution and adaptation of body functionalities may not necessarily imply a hardware change, since soft mechatronic technologies demonstrated the possibility to fulfil multiple functionalities, depending on their driving conditions (details and examples are reported in *Hardware Multifunctionality*). These three elements (soft mechatronics–The Long Way Ahead for Soft Mechatronics Development, EI through flexible design–*Embodied Intelligence by Flexible Design* and multifunctionality–*Hardware Multifunctionality*) can be combined to outline a new design approach (discussed in *Discussion*) that is prospectively able to exploit EI without formally describing design rules/guidelines.

## The Long Way Ahead for Soft Mechatronics Development

Material compliance is a key factor in soft robotics, meaning that the mechanical properties of all components need to be contextually taken into consideration. Every discontinuity in mechanical properties may determine a limitation (or even the complete failure) of the soft mechatronic approach potentiality. A soft robotics system has to be considered and designed as a single all-in-one system thus going even beyond a classic mechatronic approach in terms of integrated design. The advancement of soft robotics is thus tightly related to the improvement of bodyware technologies, both at component and at integration level. The vast majority of research on components based on soft materials is focused on actuators, but sensors, mechanisms, power supply and flexible electronics are a priority too.

### Actuation

Research on all technological aspects of soft robotics is very prolific and already achieved important milestones, although with very different technological readiness level. Soft actuation is by far the most productive topic in soft robotics and especially fluid-based soft actuators (devices based on elastomeric chamber inflated by a fluidic external source). The recent remarkable increase of studies related to this kind of actuators is well justified by the incredible potential of this approach. The underlying concept is as simple as versatile, thus it allows a very large number of possible implementations. This led to a plethora of different approaches (Gorissen et al., 2017), all relaying on the same basic principle: the volumetric deformation of the chamber caused by the fluidic pressure is driven to cause expansion towards specific directions so to result in a predefined deformation modality. However, there are many other technological approaches, that exploit different physical principle and, in some cases, have a longer and more consolidated history: shape memory materials, electro-active polymers, electro-magneto-rheological fluids and elastomers, jamming-transition based systems, low-melting-point materials, just to name the most famous (El-Atab et al., 2020).

### Sensing

EI can be used to simplify control introducing some levels of intrinsic adaptability, compensation, and reactivity mediated by the body itself with limited or no intervention from a centralized processing, but perception and active control remains very important for robots facing complex tasks. The implicit difficulty in the design of a perceptive system of a soft robot is twofold: it is necessary to take into consideration the material properties of the transducer that locally transforms the mechanical input into an electrical output (mechanosensing), and at the same time the arrangement and distribution of the sensitive units is critical as a deformation (that should potentially stimulate both the proprioceptive and exteroceptive sensing) can happen at any point of a compliant body. So far, all the main physical principles traditionally used in robotics have been translated to be fully usable in the soft robotics domain, thus we can already count on several sensing technologies based on resistive, piezoresistive, capacitive, magnetic, optical, and inductive approaches (Wang et al., 2018). The main remaining issues related to sensing and the challenges ahead are related to integration and compactness: this is attracting interest in embedding sensing functionalities into soft actuators (or other soft components) and in implementing multiple yet discriminated modality of sensing.

### Structures and Mechanisms

In robotics, structural material is usually used to support the robot weight and to determine the kinematics of body parts. Introducing compliance on structural parts completely undermines the possibility to use traditional modelling schemes, but on the other side, it enables the possibility to use compliant mechanisms, to exploit mechanical instabilities as amplification mechanisms and to develop metamaterials (e.g., origami or auxetic structures). Variable stiffness materials (such as jamming transition based or low melting point materials) can be exploited to implement variable kinematics, locking and unlocking degrees of freedom or changing body local deformability (Schmitt et al., 2018).

### Power Supply and Harvesting

Energy source is still a major issue in automation and robotics, and it is even more serious for soft systems, which have the same (or even higher) power needs and the additional limitation on the intrinsic material compliance requirements. Moreover, soft actuators and sensors currently have very limited efficiency. This is mainly due to intrinsic transduction principles (e.g., thermal as in shape memory materials) or due to the low level of technology optimization reached so far, which is rarely considering efficiency issues. Energy storage devices based on hydrogel may represent a suitable solution (Huang et al., 2021) and they may also provide alternative energy sources (fluidic, chemical and thermal) (Lee et al., 2020). Alternatively or additionally, energy could be harvested from the interaction with the environment and multiple possibilities have been already reported (Winfield et al., 2014; Pan et al., 2020).

### Logic

With the same argument introduced for sensing, EI is a powerful approach to simplify control and reduce computational burden, but it cannot substitute it, especially if the robot is expected to perform tasks with decent complexity. The presence of electric wires may not represent a major issue, especially if arranged in a way they do not mechanically interfere with motion and deformation, but other control hardware such as boards and electronic components may limit deformability. Embedding computation in soft materials would be the ultimate solution and a few attempts demonstrated the possibility to develop soft matter computers (Garrad et al., 2019), fluidic digital logic (Preston et al., 2019), and microfluidic logic circuits (Wehner et al., 2016).

## Embodied Intelligence by Flexible Design

In the context of developing a robot that has to deal with different and unknown external stimuli, the level of confidence of the designer dramatically decreases, even if the robot is designed to accomplish a limited number of tasks. Very recent advanced simulation software also based on evolutionary algorithms are becoming powerful design tools (Howard et al., 2019), but their level of abstraction is probably still not sufficient to take into account all the necessary parameters to steer the design towards the most suitable direction to deal with a real scenario (reality gap). An alternative approach could be represented by a flexible design: the integration of a certain degree of continuous adaptability in the hardware functionalities of the robot. Using a biological parallel, humans can strengthen and/or use body parts differently as for their adaptability abilities during their lifetime (ontogenetic), but body evolution is programmed by DNA and functionalities are fixed (e.g., muscles can be trained and their mass increased, but in no cases they can be turned into something with a different functionality). Functional changes happen only at evolutionary level (phylogenetic) and body functional modifications are handed down from generation to generation when the implied advantages are so significant to improve reproduction and/or survival abilities. In nature, EI is thus the outcome of evolutionary cycles. In this view, if we could loosen the necessity to design the EI of a system a priori, and only provide the body with the required elements and the ability of continuous adaptability instead, we could exploit EI principles more effectively. Continuous adaptability of hardware can be achieved with body components that are able to fulfil multiple functionalities and to establish synergies. The spatial distribution of functionalities may be left free, not because of component physical rearrangement, but due to a change of component functionality. If a relevant number of components could be embedded and their specific functionality could be set (and re-set) depending on the robot interactions and needs, it could have evolutionary capabilities in one single life cycle. As for living beings, real world would be its training ground, but functional adaptations would happen immediately (led by its current ecological niche–Pfeifer et al., 2007), and not on an evolutionary timeframe.

## Hardware Multifunctionality

In the context of flexible design introduced in the previous section, the body and its hardware components should be able to “evolve”, to adapt, changing their functionality. In *The Long Way Ahead for Soft Mechatronics Development*, all the main technological aspects of mechatronics have been debated, underlining how they have been reconsidered with a soft robotics approach and the single functionalities have been quite successfully implemented. However, there are only a few examples of studies where the underlying physical principles were used to fulfil multiple functionalities. There are three possible implementations of multifunctionalities: synergistic, static, and dynamic. Before going into details, it is worth underlining that in the first case the functionality of a component becomes coupled with another component (creating synergy), while in the other two cases the implications of the functionality change is only related to the single component.

**FIGURE 1:**
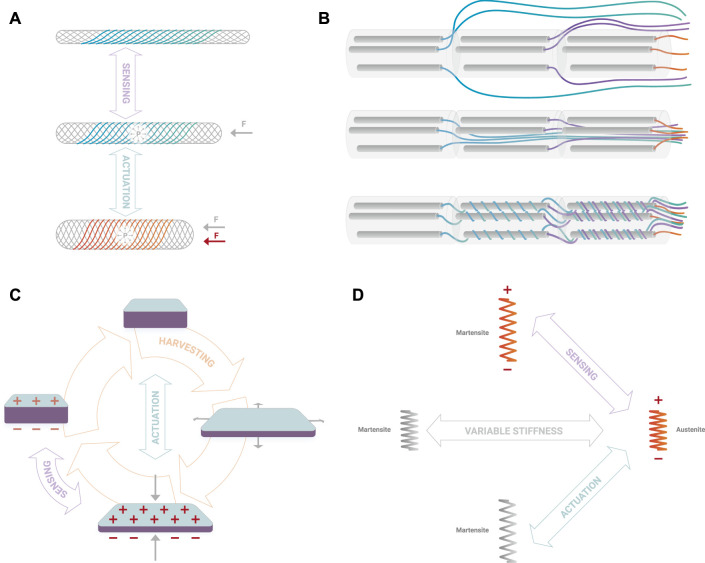
Example of technology synergistic multifunctionality **(A)** where a McKibben actuator relies on an external braided net partially made of SMA wires able to sense deformations (inactive wires in blue) or to increase the generated force (active wires in red); example of static multifunctionality **(B)** where fluid drive lines are arranged: externally leading to bulkiness **(top),** internally-straight increasing system stiffness **(centre),** or internally-helicoidal improving integration without impairing deformability **(bottom);** examples of dynamic multifunctionality depending on their driving conditions, where a Dielectric Actuator can serve as sensor, actuator or energy harvester **(C)** and a SMA can be used as sensor, variable stiffness mechanism or active actuator **(D)**.

Synergistic multifunctionality: the possibility to integrate different technologies and obtain synergies without increasing bulkiness. For example, a McKibben actuator may rely on a braided net composed of shape memory alloy (SMA) wires (Figure 1A). As long as the McKibben actuator is working normally, the SMA can be used as an integrated sensor at room temperature (exploiting inductance change, as in Felt et al., 2016) and when necessary, it could be activated through a thermal input (e.g., through Joule effect) to increase the force generated by the McKibben actuator (Daley and Abel, 2019). Other examples of technology synergies include coupling of fluidic actuators with electrostatic principles (Kellaris et al., 2018) and McKibben actuators with shape memory polymers (Takashima et al., 2010).

Static multifunctionality: a component is used to fulfil more than one functionality exploiting different intrinsic features, possibly turning potential disadvantages into advantages. For example, fluidic-driven systems usually need to deal with the presence of multiple tubes to feed the fluidic source into the actuator chamber. As the number of chambers increases, the drive lines become quite difficult to manage, especially if a distributed valve system cannot be integrated. This may become a limiting factor, impairing robot motion. The tubes are usually much harder than the elastomeric body of a soft robot to prevent the expansion of the tube itself upon inflation and they have very limited extension capabilities. An alternative solution may be to use the tubes (potential disadvantage) in helicoidal shape, designed in a way that they do not introduce a stiffness increase or may be even used as alternative to other spring elements (usable advantage). They may also cover the role of structural material for the soft robot body (Figure 1B).

Dynamic multifunctionality: to study materials or components that are able to exploit different physical principles to implement different functionalities (e.g., from actuators to sensors or power supplies) with the same embodiment. There already exist proof of technologies that fulfil multiple functions. Dielectric elastomers are usually known as active elements able to convert electrical into mechanical energy through the Maxwell stress effect, but for their nature and with no structural modifications they can be used as stretch or pressure sensors (reading the capacity variation due to induced deformation) or as accumulators (they are structurally equivalent to capacitors and thus able to store electrical charge) or energy harvesters (Figure 1C) (Thomson et al., 2018; Zhao et al., 2020). SMAs are used as active actuators exploiting their shape memory effect, but they can be also used as variable stiffness passive materials (stiffness variation induced by phase change) or as strain sensors (electrical resistance change through stress induced martensite) (Figure 1D).

These last examples are practical proof of multifunctionality and adaptability that can be introduced by design. This approach would lead to the development of extremely resilient platforms, able to cope with changes. These systems would be able to maintain effectiveness after environmental modifications, to restore damages or compensate for them, but they would also be able to dramatically change their capabilities, for instance, due to a new need. Such a robot could be re-trained (physically!) with no or very limited hardware modifications to accomplish a completely new task.

## Discussion

**FIGURE 2:**
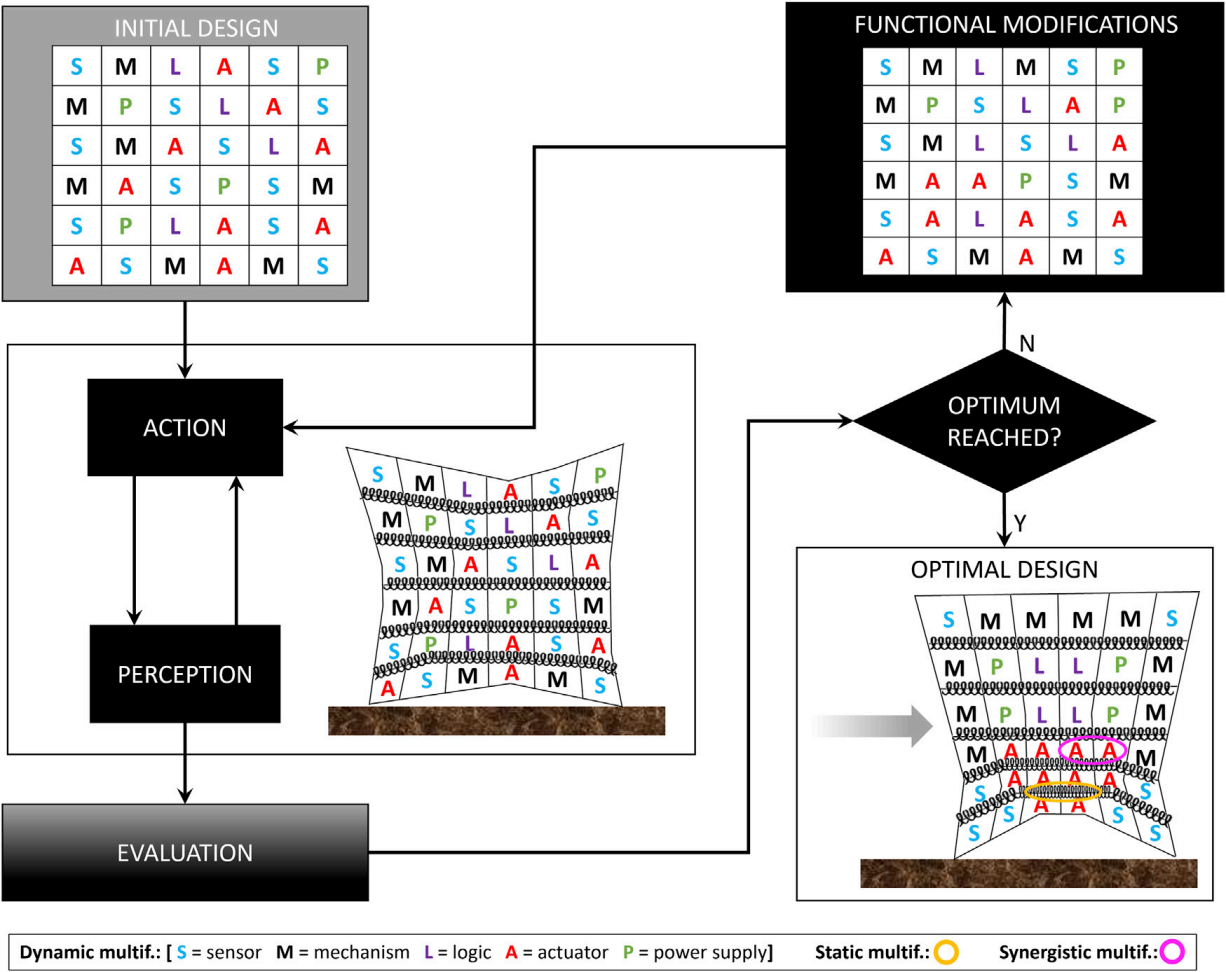
Simplified example of implementation of flexible design approach for a locomotion task. Tasks in grey blocks are expected to be executed by the designer/user. The system design can be initialized even with poor a priori knowledge, with some arbitrariness, but possibly with high redundancy; the system is then left free to interact with the environment and through action/perception loops it collects data that are used for the evaluation process (performed by the designer or possibly by the system itself); if an optimal behaviour is not reached, body components undergo functional modifications (thanks to their multifunctionality) and another iteration starts; this is repeated until an optimal behaviour is reached. Letter change indicates the change of functionality thanks to dynamic multifunctionality; fluidic tubing exploited as additional spring element (in yellow) represents a static multifunctionality; synergistic multifunctionality is highlighted in violet where actuators can couple their action.

Soft mechatronic components, EI and multifunctionality are the three main ingredients that can be combined through a flexible design approach to exploit EI principles with no guidelines and limited a priori knowledge of the environment. More concretely, the robot body may be initially built using a number of multifunctional elements set to work arbitrarily (sensors, actuators, power supply … ), also with high redundancy at the beginning. The robot could be then left free to interact with the environment and through action/perception loops it could gain experience and evolve/adapt its body (Figure 2). Multiple iterations would eventually lead to an optimal design. This process may be mediated by the designer or may be fully automatic if the designer embeds optimality targets and learning techniques that can create a relation between component functionality and overall behaviour. This does not guarantee that the process will always identify a solution able to simplify tasks (with respect to a traditional design based on the a priori available knowledge), but for sure it will be the result of the exploration of the design space led by the body interacting with the environment. Of course, the number of possible implementations is not as high as what can be achieved through simulation, but the environment is fully considered with all its peculiarities. This approach may be seen as standing in between a physical and a simulation approach. It is still based on hardware and a sort of trial-and-error process, but thanks to multifunctionality, the parts composing the body robot can assume different roles and evolve automatically without (or very limited) intervention of the user. A more detailed description of how the adaptation/evolution could actually happen in practice is still to be investigated in further research studies, but if the number of components is sufficiently high and their multifunctionality enables high functional flexibility, it is reasonable to think that (as in biological evolution) the process would be able to identify solutions where the body is used as a resource to simplify task execution and in making adaptive behaviours emerge through the interaction with the environment.

## Conclusion

Soft robotics has undoubtedly produced a series of new concepts and technologies that is vastly expanding the potential impact of robotics in our society. Its theoretical advantages related to intrinsic safety, redundancy, dexterity have been demonstrated and successfully exploited in some specific areas such as the food industry (in grippers for delicate manipulation–Hughes et al., 2016; Zaidi et al., 2021) and the biomedical sector (especially in the implementation of biomimetics and bioinspired approaches) (Ashuri et al., 2020; Cianchetti et al., 2018), but no application with a revolutionary impact has been clearly demonstrated so far. Despite these valuable examples, indeed, the soft robotics community is concerned about the future of this discipline. One of the most used metrics to quantify its impact is represented by the number of publications that use the term “soft robotics”, and it is experiencing an exponential growth. This emphasis and resonance are now justified, as a young discipline offers several investigation paths and it gives the opportunity to push some missing technologies forward. However, going beyond terminology, the game changing idea is the awareness of the importance of mechanical compliance and the efforts to exploit it IF and WHERE needed. Thus, in the near future, this distinction may not be useful anymore as soft robotics could become integral part of robotics at large. Maybe, we will think about soft robotics as the historical period when roboticists started to look at softness and variable compliance as means to build better robots and enrich their behaviour. We may stop speaking about “soft robots”, but we will have many robots based on soft robotics principles. In any case, efforts should be focused on understanding how to unleash the main essence of soft robotics that can fully exploit material complexity. Several ingredients are available, but we are still missing the right recipe and tools to master and combine them effectively. A flexible design approach could be a further step forward in the implementation of EI principles exploiting multifunctionality. This may represent the added value for soft robotics to follow a thriving and impactful trajectory on the next decade (Hawkes et al., 2021) and to reach a real contribution to robotics and engineering more broadly.

## Data Availability

The original contributions presented in the study are included in the article/Supplementary Material, further inquiries can be directed to the corresponding author.
